# Epidemiology of viral and atypical bacterial respiratory infections among children hospitalised at a Kenyan tertiary referral centre: a cross sectional multiplex PCR study

**DOI:** 10.3389/fped.2026.1792031

**Published:** 2026-07-23

**Authors:** Rohini Kalagouda Patil, Anne Irungu, John Chelanga Supra, Rahul Zode, Koigi Paul Kamau, Joseph K. Mbuthia, Diana Marangu-Boore

**Affiliations:** 1 Department of Paediatrics, The Nairobi Hospital, Nairobi, Kenya; 2 College of Health Sciences, The Nairobi Hospital, Nairobi, Kenya; 3 Department of Laboratory Medicine, The Nairobi Hospital, Nairobi, Kenya; 4 Department of Medical Research, The Nairobi Hospital, Nairobi, Kenya; 5 Department of Paediatrics and Child Health, University of Nairobi, Nairobi, Kenya

**Keywords:** Africa, rapid molecular testing, paediatric, pneumonia, seasonality, viruses

## Abstract

**Background:**

Severe acute respiratory infections (SARIs) cause high paediatric morbidity and mortality particularly in sub-Saharan Africa. Understanding contemporary trends in SARI aetiology is needed to inform prevention strategies. We aimed to determine the current aetiology, severity and seasonality of SARIs based on pathogens detected by multiplex quantitative real-time polymerase chain reaction (qRT-PCR) tests, BioFire® respiratory panel 2.0 (RP 2) and 2.1*plus* (RP2.1*plus*)

**Methods:**

We conducted a retrospective cross-sectional analytic study among Kenyan children aged 0–18 years hospitalised between March 2018 and March 2023, with SARI and a positive nasopharyngeal swab multiplex qRT-PCR test. Cases were analyzed for their clinical characteristics, radiological features, laboratory data, seasonality of the respiratory pathogens and risk factors for severe infection.

**Results:**

A total of 545 pathogens were detected in 382 cases,123 (22.5%) had polymicrobial detection, 14 (4%) had severe illness and three (0.8%) died. Rhinovirus (24%) and respiratory syncytial virus (21%) were the most common viral pathogens identified whereas *Mycoplasma pneumoniae* (2.4%) and *Bordetella pertussis* (1.8%) were the most common atypical pathogens detected. Infections peaked during the first dry season and the short rainy season. Lower respiratory tract infections and presence of co-morbidities like asthma,vitamin D deficiency and iron deficiency anaemia were associated with disease severity independent of age, sex and seasonality.

**Conclusions:**

Viruses accounted for majority of respiratory infections. Infections had two distinct peaks: the first dry season and the short rainy season, suggesting that respiratory vaccines should be scheduled prior to their onset.

## 1 Introduction

Lower respiratory tract infections (LRTIs) cause significant morbidity and mortality in sub-Saharan Africa, especially in children (1). Data from sub-Saharan Africa show that childhood pneumonia is caused by a relatively limited group of respiratory pathogens, with respiratory syncytial virus (RSV) consistently identified as a leading cause, while *Streptococcus pneumoniae*, *Haemophilus influenzae*, and in some settings *Mycobacterium tuberculosis* remain important contributors. *Streptococcus pneumoniae and Haemophilus influenzae type b (Hib)* cases have reduced due to the impact of vaccines, with *Staphylococcus aureus and* non-typeable *H. influenzae* being the leading bacterial pathogens in low-and-middle-income-countries (LMICs). The Pneumonia Etiology Research for Child Health (PERCH) Study and related African studies from The Gambia, Kenya, Zambia, and South Africa have substantially advanced understanding of pneumonia etiology in the post-*Hib* and pneumococcal conjugate vaccine era. Findings from South Africa, Kenya, and Mozambique similarly underscore the major contribution of viral pathogens, particularly RSV, to severe childhood respiratory illness in sub-Saharan Africa (2–5). Respiratory infections tend to be perennial but have been noted to peak in the rainy season in Kenya^1^ (6, 7).

Although the empiric use of antibiotics for pneumonia is guided by global and national guidelines, there is high value in generating robust evidence to inform these guidelines. The World Health Organization (WHO) defines severe acute respiratory infections (SARIs) as symptoms less than ten days, cough, fever above 38 °C and hospitalization (8). Our Kenya national paediatric guidelines classify the severity of pneumonia based on oxygen need and other systemic clinical signs to guide empiric treatment^2^.

Molecular tests like multiplex quantitative real-time polymerase chain reaction (qRT-PCR) are useful, rapid and highly sensitive for the diagnostic work-up of respiratory infections. This test can detect common bacterial, viral and atypical pathogens and be utilised in addition to other markers of bacterial infection, to guide usage of antibiotics. It plays a role in guiding infection control and objectively defining the duration of isolation measures for contagious pathogens like influenza virus and severe acute respiratory syndrome coronavirus-2 (SARS-CoV-2). It also provides reliable data capable of defining the seasonality of pathogens that may inform vaccination strategies (9–11).

The qRT-PCR test is expensive and not widely available locally. The few studies that have been done in Kenya on qRT-PCR utility in children for respiratory infections have mainly been epidemiological, not for routine clinical use (2, 5, 7). Our study aimed to decribe the clinical, laboratory, radiological charcteristics, seasonality of the respiratory pathogens detected and to determine the factors associated with disease severity based on the pathogens detected by multiplex quantitative real-time polymerase chain reaction (qRT-PCR) tests, BioFire® respiratory panel 2.0 (RP 2) and 2.1*plus* (RP2.1*plus*). The findings of this study may inform other clinicians and institutions in similar settings, regarding the aetiological diagnosis and treatment of children admitted with common respiratory infections especially during seasonal outbreaks.

## 2 Methods

### 2.1 Study design

We conducted a retrospective cross-sectional analytic study between March 2018 to March 2023 among children from birth to less than eighteen years hospitalised with acute respiratory infections with a positive BioFire® respiratory panel 2.0 (RP 2) and 2.1*plus* (RP2.1*plus*) test performed on a nasopharyngeal sample.

### 2.2 Objectives

Our primary objectives were to describe clinical, laboratory and radiological characteristics of children included in the study, and seasonality of the respiratory pathogens. Our secondary objective was to determine the factors associated with disease severity.

### 2.3 Data collection

We included children hospitalised with acute respiratory infections (ARIs) based on a clinician's diagnosis reported as either an upper or lower respiratory tract infection or both as per WHO ICD-10 classification^3^. Those with incomplete clinical biodata or who had a respiratory BioFire® test performed as an outpatient were excluded.

### 2.4 Study size

We used the Cochran formula to estimate the sample size based on a similar study (9, 12). A minimum sample size of 306 was calculated.

Detailed information on patient demographics, clinical characteristics, vital signs, clinical diagnosis, laboratory parameters and radiological findings on plain chest radiography were recorded. Abnormal radiological features were those suggestive of lower respiratory tract infection in the form of consolidation, infiltration, infiltrates, opacities, atelectasis, pleural effusion, pneumonia, pneumonitis or bronchiolitis. Weight for age and body mass index for age z-scores were used to determine nutritional status.

### 2.5 Variables

The dependent variables were the severity of the disease (mild, moderate and severe). Mild cases were those without hypoxemia (oxygen saturation above 92%) and did not require oxygen supplementation. Moderate cases were defined as those that required low-flow oxygen therapy either by nasal prongs, a simple facemask, or a non-rebreather mask. Severe cases were those that required high oxygen flows up to 60 litres/min using either high-flow nasal cannula, invasive or non-invasive ventilation; and were admitted to the paediatric intensive care unit (PICU) or high dependency unit.

Based on the site, infections were dichotomized into upper and lower respiratory tract infection. Upper respiratory tract infections included tonsillitis, pharyngitis, rhinitis, adenotonsillitis, and otitis media; and lower respiratory tract infections comprised pneumonia, bronchiolitis, bronchitis and pneumonitis. The presence of any attendant comorbidities like sickle cell disease, malnutrition, gastroesophageal reflux, chronic renal, hepatological, neurological or cardiac conditions, vitamin deficiencies among others were also recorded.

The independent variables were age, gender, nutritional status, type of respiratory infection, comorbidity, level of care based on severity, radiological features, duration of hospital stay less or more than 7 days and outcomes of severity in the form of death or discharge home. We also recorded complications like sepsis and/or shock, pleural effusion, pneumothorax, empyema and oxygen dependency on hospital discharge.

### 2.6 Laboratory methods

Appropriate laboratory testing on blood, sputum and urine was conducted on certain patients based on the clinical presentation. Normal laboratory ranges for white cell count, C-reactive protein and procalcitonin were 5.5–15.5 × 10^9^/L, 0–5 mg/L and 0–2.0 ng/mL respectively, any values outside the above range was considered abnormal (Supplementary Table S1). A nasopharyngeal specimen was collected and processed according to the standard technique on the FILMARRAY® instrument for BioFire® Respiratory Panel 2.0 (RP2.0) and BioFire® RP2.1*plus* (BioFire® Diagnostics, bioMérieux, France). The FILMARRAY® instrument is an automated closed system with a disposable pouch for sample preparation, isolation, amplification and detection of nucleic acid from multiple respiratory pathogens within a single nasopharyngeal swab specimen. The entire run process takes about 45 min. The BioFire® Respiratory Panel 2.0 (RP2.0) can detect 17 viruses and four atypical bacteria that cause respiratory tract infections with an overall sensitivity and specificity of 95.8% and 99.7% respectively. The BioFire® RP2.1 *plus* in addition, detects Middle East respiratory syndrome coronavirus (MERS-CoV) and SARS-CoV-2 with a sensitivity of 97.1% and a specificity of 99.3%. The BioFire® Respiratory panel 2.0 (RP 2.0) was utilized up to the year 2021 and thereafter BioFire® Respiratory panel 2.1 *plus* (RP2.1 *plus*) was used (Supplementary Table S2).

### 2.7 Data analysis

We analysed our data using Statistical Package for Social Sciences Version 25.0 (SPSS®, IBM corp, USA). Categorical data were presented as frequencies and percentages; and continuous data summarized as medians with interquartile intervals. Pearson Chi-square test was used to determine the strength of association between patient characteristics and severity of the respiratory infection at 95% significance. The seasonal variation of the respiratory pathogens was presented using seasonal charts. The seasons were divided into the first dry season from 15_th_ December to 30th of March, the long rainy season from 1st April to 30th June, the second dry season from 1st July to 31st October and the short rainy season from 1st of November to 15th of December.

Age, sex, and comorbidities were considered potential confounding factors a priori and included in the multivariable logistic model. Severe disease was the outcome of interest; mild and moderate cases were combined and classified as non-severe for comparison. Multiple logistic regression was used to measure the strength of association between the predictors and severity of respiratory tract infection. Prior to analysis, the assumptions for the model on independence of the predictors, absence of multicollinearity and outliers were assessed. At analysis, adequacy of the model was assessed using Likelihood ratio test. Our model was a significant fit.

## 3 Results

**Figure 1:**
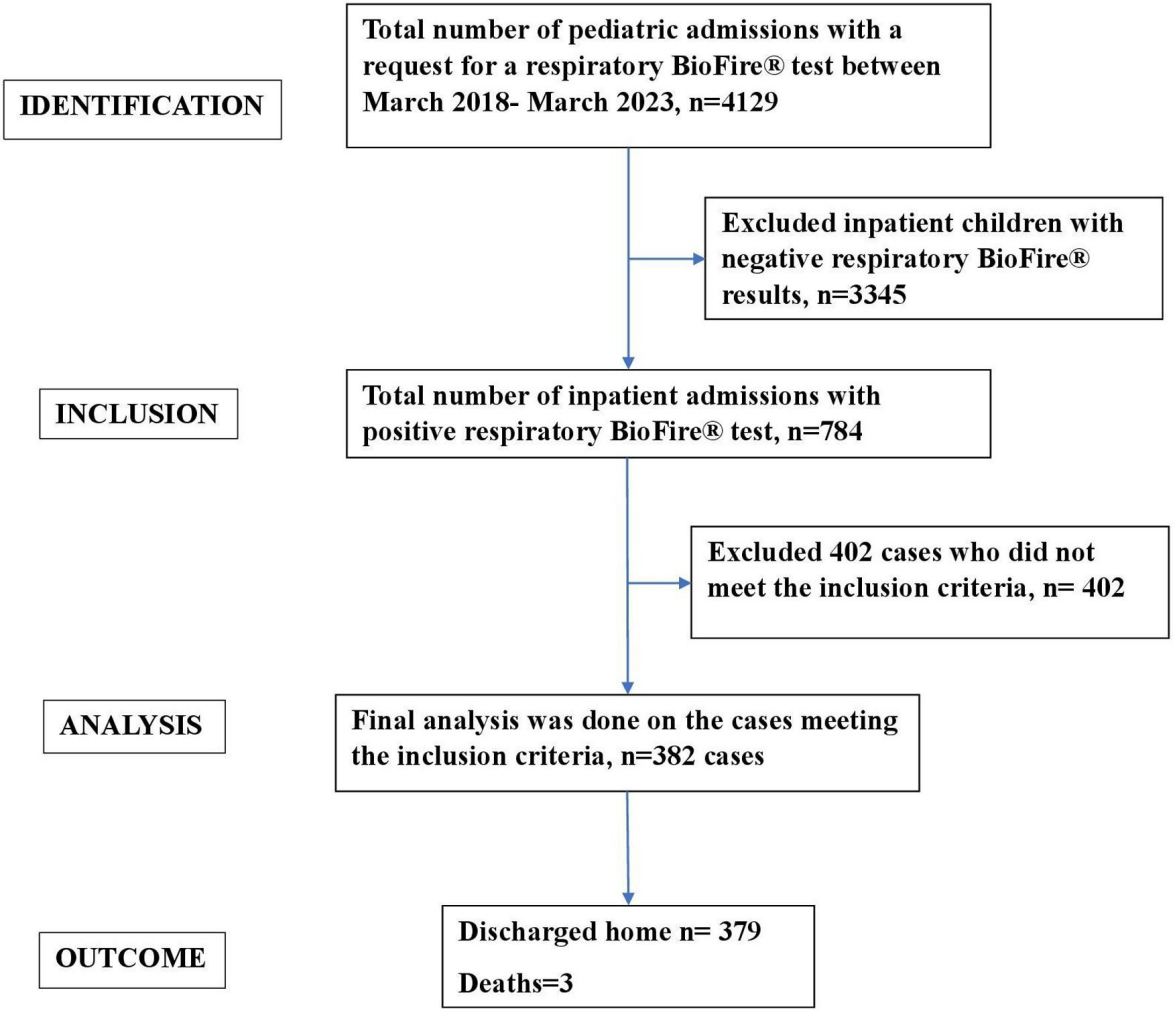
STROBE flow chart of cases with acute respiratory tract infection amongst paediatric patients with respiratory pathogens detected by BioFire® Respiratory panel 2.0 (RP 2) and 2.1plus (RP2.1 *plus*) test admitted between March 2018 and March 2023 at a Kenyan tertiary hospital.

A total of 4,129 hospitalised cases between March 2018 to March 2023 were tested. There were 784 cases with a positive respiratory BioFire® test, 382 of whom met the inclusion criteria (Figure 1). A total of 545 organisms were detected on PCR testing in 382 cases of which 514 (94.3%) were viral and 31 (5.7%) atypical bacteria. A total of eleven bacterial cultures: six sputum, three blood and two urine isolates were reported positive. The patient characteristics are summarized in (Table 1).

**Table 1:** Characteristics of paediatric patients with respiratory pathogens detected by bioFire® respiratory panel 2.0(RP2.0) and 2.1*plus* (RP2.1*plus*) test hospitalised between March 2018 and March 2023 at a Kenyan tertiary hospital, based on the severity of the respiratory infection.

Variable	Severity of respiratory infection				
Age	Mild (*n* = 276) n (%)	Moderate (*n* = 92) n (%)	Severe (*n* = 14) n (%)	Total (*N* = 382) n (%)	*p*-value
<1 month	0 (0)	5 (1.3)	2 (0.5)	7 (1.8)	<0.001
2–11 months	65 (17.0)	37 (9.6)	4 (1)	106 (27.7)	
1–5yrs	165 (43.2)	45 (11.8)	5 (1.3)	215 (56.3)	
6–10yrs	38 (9.9)	4 (1.0)	2 (0.5)	44 (11.5)	
>10yrs	8 (2.1)	1 (0.2)	1 (0.2)	10 (2.6)	
Gender					
Male	141 (36.9)	59 (15.4)	9 (2.3)	209 (54.7)	0.061
Female	135 (35.3)	33 (8.6)	5 (1.3)	173 (45.3)	
URTI					
Yes	175 (45.8)	31 (8.1)	2 (0.5)	208 (54.4)	<0.001
No	101 (26.4)	61 (15.9)	12 (3.1)	174 (45.5)	
LRTI					
Yes	199 (52.1)	85 (22.3)	12 (3.1)	296 (77.4)	<0.001
No	77 (20.2)	7 (1.8)	2 (0.5)	86 (22.6)	
Comorbidity					
Yes	109 (28.5)	44 (11.5)	10 (2.6)	163 (42.7)	0.036
No	167 (43.7)	48 (12.6)	4 (1.0)	219 (57.3)	
Level of care					
HDU	0 (0.0)	1 (0.3)	0 (0.0)	1 (0.3)	<0.001
PICU	2 (0.5)	9 (2.4)	13 (3.4)	24 (6.3)	
Ward	274 (71.7)	83 (21.7)	0	357 (93.4)	
Radiological features					
Absent	57 (14.9)	18 (4.6)	2 (0.7)	77 (20.2)	0.19
Present	134 (35.1)	69 (18.1)	11 (2.8)	214 (56.0)	
Not done	85 (22.3)	5 (1.2)	1 (0.3)	91 (23.8)	
Nutritional status					
Healthy	143 (37.4)	47 (12.3)	8 (2.1)	198 (51.8)	0.491
Overweight	45 (11.8)	9 (2.3)	3 (0.8)	57 (14.9)	
Underweight	39 (10.2)	16 (4.2)	2 (0.5)	57 (14.9)	
Missing data	49 (12.8)	20 (5.2)	1 (0.3)	70 (18.3)	
Duration of stay					
<7 days	205 (53.7)	51 (13.3)	1 (0.2)	257 (67.3)	<0.001
>7 days	71 (18.5)	41 (10.7)	13 (3.4)	125 (32.7)	
Outcome					
Mortality	1 (0.003)	0 (0)	2 (0.14)	3 (0.007)	
Discharged home	275 (72)	92 (24.1)	12 (3.1)	379 (99.2)	< 0.001

URTI, upper respiratory tract infections; LRTI, lower respiratory tract infections; HDU, high dependency unit; PICU, paediatric intensive care unit. *p*-values by Chi^2^ for binary/categorical variables.

Out of the total 382 cases, 276 (72.3%) had mild, 92 (24%) moderate and 14 (3.7%) cases were classified in the severe category. Almost two thirds of the 312 cases with complete nutritional data had healthy weight, 198 (63.5%).

### 3.1 Clinical characteristics

Comorbidities were noted in 163 (42.7%) cases, some cases had more than one comorbidity at admission. Asthma was noted in 51 (31%), 31 (19.1%) had vitamin D deficiency and 27 (16.6%) iron deficiency anaemia. Neurological cases which included; Cerebral palsy, seizures, convulsions, encephalopathy, acute disseminated encephalomyelitis, Guillain-Barre syndrome and epilepsy diagnoses, were 16 (9.8%). Gastroesophageal reflux disease were 13 (8.0%), 12 (7.4%) adenoid hypertrophy and 9 (5.5%) allergic rhinitis cases. Sickle cell disease, Human immunodeficiency virus, Trisomy 21 and malignancies had 2 (1.2%) cases each. Only 1 (<1%) cardiac and a preterm with chronic lung disease case were noted.

### 3.2 Laboratory findings

**Figure 2:**
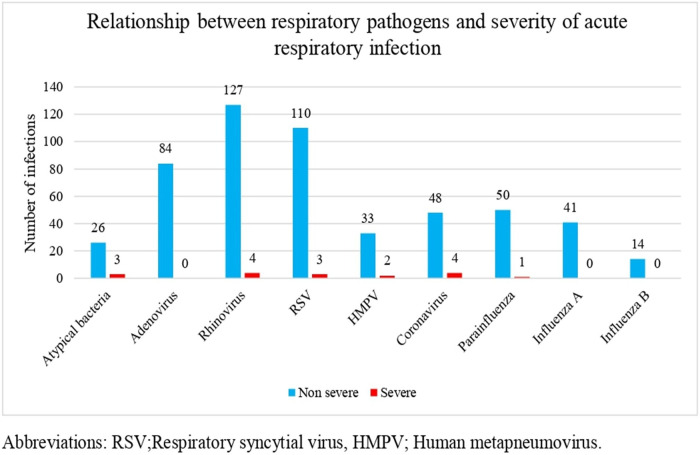
Respiratory pathogens and severity of presentation of SARI amongst paediatric patients with respiratory pathogens detected by BioFire® Respiratory panel 2.0 (RP2) and 2.1plus (RP2.1 *plus*) test admitted between March 2018 and March 2023 at a Kenyan tertiary hospital.

Human rhinovirus/enterovirus was the most prevalent pathogen with 131 (24.0%) infections (Figure 2). In infancy, 147 viruses and 9 atypical bacteria were identified. RSV was the most commonly detected pathogen with 42 infections noted (27%) (Supplementary Table S3). Multiple microbial detection (co-detection) occured in 123 (22.5%) patients, (Supplementary Table S4).

Atypical bacterial infections were noted in 31/545 (5.7%) PCR tests and were more prevalent between one month to five years of age, 23/31 (74.2%). Among atypical bacteria, *Mycoplasma pneumoniae* was the commonest with 13/31 (41.9%) infections, followed by *Bordetella pertussis* 10/31 (32.3%), *Bordetella parapertussis* 5/31 (16.1%) and *Chlamydophila pneumoniae* 3/31 (9.7%). *M. pneumoniae* was more common in children less than five years old, 9/13 (69.2%) (Supplementary Table S3).

Sputum cultures were performed on nine patients, six of which (66.7%) were positive. *Klebsiella pneumoniae*, *Streptococcus viridans* and *Pseudomonas aeruginosa* had two isolates each. Blood culture specimens were 31 and three (9.7%) were positive; two *Coagulase negative Staphylococcus* isolates, and one *Staphylococcus aureus* isolate. Only two of 19 (10.5%) urine culture samples were positive, *K. Pneumoniae* and *Morganella morganii* respectively. No significant statistical association was noted between severity and the haematologic parameters, liver and kidney function tests, C-reactive protein or pro-calcitonin (Supplementary Table S5, S6, S7).

A total of 381 cases underwent laboratory testing for hemogram. Elevated white cell counts(WBCs) were noted in 90(23.6%) and 36(9.4%) had low WBCs. However, the relationship between patient's WBCs count and severity of the respiratory infection was not statistically significant (*p* = 0.315). However, 57.1% of SARI cases with severe presentation had low lymphocyte counts in our study. However, the association between lymphocyte count and severity of the respiratory tract infections was not conclusive (*p* = 0.573). A total of 359 cases underwent C -reactive protein(CRP) testing, 212 (59.1%) had elevated CRP level. The association between CRP levels and severity of the respiratory infection was not statistically significant (*p* = 0.602). Of the 143 cases who underwent procalcitonin testing, Elevated procalcitonin level was observed in 21(14.7%) patients with mild infection, 8(5.6%) patients with moderate infection and one (0.7%) with severe infection (*p* = 0.994). See Supplementary Tables S5, S6 and S7 for laboratory investigations.

#### 3.3 Radiological findings

Chest radiographs were performed in 291/382 (76.2%) cases, of which 214/291 (73.5%). Normal radiological features were seen in 77 (26.4%) of the patients.Those with normal radiological features, 57 (74.0%) had mild infection and 18 (23.3%) had moderate infection. Abnormal radiological findings were seen in 134 (62.6%) patients with mild infection, 69 (32.2%) with moderate infections and 11 (5.14%) with severe infections. Majority (78.5%) of the patients with severe respiratory infection had radiological features consistent with lower respiratory infections. Abnormal radiological features were associated with severity of respiratory infection (*p* = 0.037).

#### 3.4 Seasonality

**Figure 3:**
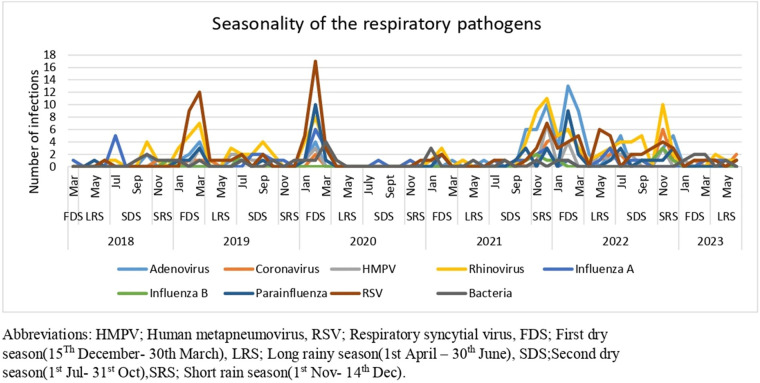
Seasonality of the respiratory pathogens of cases with acute respiratory tract infection amongst paediatric patients with respiratory pathogens detected by BioFire® Respiratory panel 2.0 (RP2) and 2.1 plus (RP 2.1 *plus*) test admitted between March 2018 and March 2023 at a Kenyan tertiary hospital.

Both viral and atypical bacterial infections were perennial. The first dry season had the highest peak of infections at 233/545 (42.7%) infections followed by the short rainy season with 134 (24.6%) infections, the second dry season 111 (20.4%) and lastly the long rainy season with 67 (12.3%) cases as depicted in (Figure 3 and Table 2).

**Table 2:** Seasonal prevalence of respiratory pathogens detected by bioFire® respiratory panel 2.0 (RP 2) and 2.1*plus* (RP2.1*plus*) test amongst hospitalised paediatric patients.

Pathogen count	First dry season *N* = 233	Long rain season *N* = 67	Second dry season *N* = 111	Short rainy season *N* = 134	Total infections *N* = 545
Adenovirus	38 (6.9)	6 (1.1)	15 (2.7)	25 (4.6)	84 (15.4)
Corona Virus 229E	1 (0.2)	0	1 (0.2)	0	2 (0.4)
Corona Virus HKU1	1 (0.2)	0	1 (0.2)	0	2 (0.4)
Corona Virus OC43	3 (0.6)	4 (0.7)	3 (0.6)	4 (0.7)	14 (2.6)
Corona Virus NL63	4 (0.7)	1 (0.2)	0	2 (0.4)	7 (1.3)
Severe acute respiratory syndrome coronavirus 2 (SARS-CoV-2)	9 (1.7)	3 (0.5)	1 (0.2)	7 (51.3)	20 (3.7)
Human Metapneumovirus	12 (2.2)	5 (0.9)	8 (1.5)	10 (71.8)	35 (6.4)
Human Rhinovirus/ Enterovirus	49 (8.9)	14 (2.6)	33 (6.0)	35 (6.4)	131 (24)
Influenza A	0	1 (0.2)	0	0	1 (0.2)
Influenza A/H1	0	1 (0.2)	1 (0.2)	0	2 (0.4)
Influenza A/H 1–2009	13 (2.4)	4 (0.7)	9 (1.6)	1 (0.2)	27 (4.9)
Influenza A/H3	1 (0.2)	1 (0.2)	3 (0.5)	6 (1.1)	11 (2.0)
Influenza B	1 (0.2)	0	4 (0.7)	9 (1.6)	14 (2.6)
Para Influenza 1	1 (0.2)	1 (0.2)	0	3 (0.5)	5 (0.9)
Para Influenza 2	1 (0.2)	3 (0.5)	4 (0.7)	1 (0.2)	9 (1.6)
Para Influenza 3	19 (3.5)	3 (0.5)	5 (0.9)	1 (0.2)	28 (5.1)
Para Influenza 4	5 (0.9)	0	1 (0.2)	3 (0.5)	9 (1.6)
Respiratory Syncytial Virus(RSV)	64 (11.7)	16 (2.9)	15 (2.7)	18 (3.3)	113 (20.7)
*Bordetella pertussis*	4 (0.7)	0	4 (0.7)	2 (0.4)	10 (1.8)
*Bordetella parapertussis*	1 (0.2)	0	0	4 (0.7)	5 (0.9)
*Chlamydophilia pneumoniae*	0	2(0.4)	1(0.2)	0	3(0.5)
*Mycoplasma pneumoniae*	6(1.1)	2(0.4)	2(0.4)	3(0.5)	13(2.4)

SARS COV-2, severe acute respiratory syndrome Coronavirus 2; RSV: respiratory syncytial virus. a total of 545 organisms were detected on the multiplex PCR.

#### 3.5 Cases with severe clinical presentation

A total of 14 (3.7%) patients had clinically severe illness. Two deaths (14.2%) occurred in this group. Most of the cases had comorbidities, 13(93%). Neurological conditions were the most common of the comorbidities, noted in 7 cases. All severe cases were admitted in PICU with a prolonged hospital stay of more than seven days. Rhinovirus (4) and RSV (3) were the most common pathogens. *B.pertussis* was the only atypical bacteria isolated in this group. Seasonality of infection was similar to the rest of the group, but the organisms differed, as adenovirus and influenza virus were not observed. The most common complication noted was sepsis in four (28.6%) cases (Table 3).

**Table 3:** Characteristics of hospitalised children with respiratory infections detected by bioFire® respiratory panel, in the severe illness group.

Variable	*N* = 14	(%)
Seasonality		
First Dry	5	35.7
Long Rainy	2	14.3
Second Dry	4	28.6
Short Rainy	3	21.4
Comorbidities		
Cerebral Palsy/Convulsions	4	28.6
Epilepsy	2	14.3
Guillain Barre Syndrome	1	7.1
Down's Syndrome	2	14.3
Prematurity/ Chronic Lung Disease	1	7.1
Oxygen Requirement		
Continuous Positive Airway Pressure Ventilation (CPAP)	8	57.1
Mechanical Ventilation	6	42.9
Level Of Care		
Intensive Care Unit	14	100
Organism Detection		
Single	12	85.7
Multiple	2	14.3
Organisms		
Rhinovirus	4	28.6
Coronavirus OC43	2	14.3
SARS-CoV-2	2	14.3
Respiratory syncytial virus	3	21.4
*Bordetella Pertussis*	2	14.3
Human Metapneumovirus	2	14.3
Parainfluenza 3	2	14.3
Complications		
Sepsis	4	28.6
Hyperglycemia	1	7.1
Multiple Infections (Co-detections)		
Rhinovirus, Parainfluenza-3, Respiratory Syncytial Virus	1	7.1
Parainfluenza-3 and Respiratory Syncytial Virus	1	7.1

#### 3.6 Predictors of severity

A clinical diagnosis of lower respiratory tract infection, AOR [6.4, 95%CI (1.4, 29.2)] and the presence of comorbidities, AOR [3.5, 95%CI (1.18;11.4)] were significantly associated with disease severity, independent of age, sex and seasonality (Table 4).

**Table 4:** Unadjusted and adjusted odds ratios for factors associated with severity of respiratory infection using multivariable logistic regression model.

Variable	Non-severe cases	Severe	Unadjusted Odds Ratio		Adjusted Odds Ratio		*p*-value
Age	(*n* = 368)	(*n* = 14)	OR	95% CI	AOR	95% CI	
0–1 year	107 (28%)	6 (1.6%)	0.50	0.06; 4.67	0.47	0.31;1.56	0.21
1–5 years	210 (54.9%)	5 (1.3%)	0.21	0.02; 2.01	1.01	0.82; 5.61	0.99
6–10 years	42 (10.9%)	2 (0.5%)	0.43	0.04; 5.25	1.43	0.14; 15.17	0.77
>10 years	9 (2.4%)	1 (0.2%)	Reference		Reference		
Gender							
Female	168 (43.9%)	5 (1.3%)	0.65	0.22; 1.99	1.78	0.57; 5.60	0.32
Male	200 (52.4%)	9 (2.4%)	Reference		Reference		
Respiratory infections							
LRTI (Present)	284 (74.3%)	12 (3.14%)	2.04	1.87; 8.03	6.36	1.38; 29.24	0.02
LRTI (absent)	84 (21.9%)	2 (0.5%)	Reference		Reference		
Comorbidity							
Present	153 (40%)	10 (2.6%)	3.51	1.08; 11.39	3.51	1.18; 11.39	0.03
Absent	215 (56.2%)	4 (1%)	Reference		Reference		
Organisms							
Multiple organisms	121 (31.6%)	2 (0.5%)	2.90	0.67; 13.56	2.89	1.67; 6.39	0.03
Single organism	247 (64.6%)	12 (3.14%)	Reference		Reference		
Seasonality							
First dry	158 (41.3%)	5 (1.3%)	0.88	0.20; 3.76	0.87	0.20; 3.81	0.88
Long rainy	48 (12.5%)	2 (0.5%)	1.17	0.19; 7.23	0.76	0.12; 4.94	0.77
Second dry	78 (20.4%)	4 (1%)	1.41	0.31; 6.54	1.56	0.33; 7.75	0.57
Short rainy	84 (21.9%)	3 (0.8%)	Reference		Reference		

CI, confidence interval; LRTI, lower respiratory tract infection; URTI, upper respiratory tract infection.

#### 3.7 Mortality

Three mortalities occurred in our study population, two females both aged five years and one male aged three years. Comorbidities associated with them were a brain tumour with raised intracranial pressure, prematurity with chronic lung disease, and Down's syndrome with epilepsy respectively. They all had single organism infection with *B. pertussis*, human metapneumovirus and RSV respectively. The child with a brain tumour had minimum requirement of oxygen (mild severity) and was admitted in the paediatric intensive care unit for acute management of seizures.

## 4 Discussion

In this study, majority of cases were mild and mostly afflicted those less than five years of age. Males were affected more than females in concurrence with other similar studies (9, 13). The presence of a lower respiratory tract infection and comorbidities were associated with clinical severity. Most of the pathogens were viral, 514 (94.3%) and showed mostly two seasonal peaks during the year.

The common comorbidities observed in this study were asthma, vitamin D deficiency, neurological disorders and iron deficiency anaemia, similar to that noted by Marangu and Zar's update on childhood pneumonia (3). A recent systemic review and meta-analysis revealed that chronic conditions among children aged 6 to 24 months enhanced the propensity towards developing influenza-related acute LRTI (14).

Viruses are the typical etiologic agents of ARI in children, especially after the introduction of pneumococcal and *Hib* vaccinations (2–4, 9). The commonest viruses encountered in our region before COVID-19 era were human coronavirus, influenza-A, parainfluenza-3, adenovirus, and metapneumovirus (2–4), which did not change significantly during the COVID-19 pandemic (10, 11, 15). RSV infection was high among the infants and under 5 years old, similar to previous local studies (2, 4).

Co-detections were not high in our group in 123 (22.5%) patients, and only 2 out of 14 (14.3%) patients in the severe group had co-detections. Co-detections with respiratory viruses, such as RSV, bocavirus (BoV) and SARS-CoV-2 have been shown to increase the severity of the disease in children with ARI (15, 16). Incidence of co-detections with either bacteria and virus combination or multiple viral co-detections have been reported with varying outcomes (17–20).

Among the atypical bacteria, *M. pneumoniae and B. pertussis* were the most frequently detected. Although the incidence of pertussis in East Africa reduced after the mass whole cell vaccination, we noted 6 of the 10 *pertussis* cases in our cohort, in children above 12 months old (Supplementary Table S3). This is despite a high three dose *pertussis* vaccine coverage of 89%, noted in the national health survey done in 2022 in this age group^4^. This may be due to possibility of some vaccine ineffectiveness and immunity waning as they approach five years (21).The predominance of *M. pneumoniae* cases in children under 5 years of age in our cohort should be interpreted cautiously given the small number of *M. pneumoniae* cases identified and larger studies are needed to better define the age distribution in our population.

Although culture is the gold standard to isolate *Mycoplasma* and *Bordetella* species, PCR has been shown to have a higher sensitivity and improved yield (22). Typical bacterial pathogens are not unusual in our region, however, the molecular panel used in this study does not include typical bacterial pathogens (Supplementary Table S2) (2, 4).

Blood and sputum cultures were not performed on all moderate to severe cases which limited our analysis, especially of the typical community acquired pathogens.*Coagulase negative Staphylococcus and Streptococcus viridans* detected in blood and sputum cultures are commensals, thus were probably contaminants and were noted in mild cases. *K. pneumoniae, S. aureus and P. aeruginosa* belong to the ESKAPE (group of six highly virulent, multi-drug resistant bacteria—E*nterococcus faecium, S. aureus, K. pneumoniae, Acinetobacter baumannii, P. aeruginosa, and Enterobacte*r *spp*.) associated with nosocomial and community acquired infections like in our study (2, 3, 23).

Laboratory studies were not significantly associated with the severity of the disease in our study (Supplementary Table S5,S6,S7). Some studies however did note leukocytosis and neutrophilia associated with ADV and HRV causing severe disease (24). Han et al. (25) noted that Neutrophil-to-lymphocyte ratio and low platelets had high sensitivity in screening for IFV infections, SARS-CoV-2 and severe COVID −19 was associated with continuous decline in the hemoglobin level and thrombocytopenia. Lymphocytes were related to the entire course of ADV infection, recovery of COVID-19 and disease development of SARS (26). Mean corpuscular hemoglobin concentration(MCHC) could also be good predictors of SARIs (27).

Although mortality was low in our cohort at less than one percent, all three fatalities had co-morbidities. Prematurity is associated with significant respiratory morbidity including chronic lung disease, risk of severe viral bronchiolitis and mortality (28, 29). Metapneumovirus is associated with mortality in children with complex chronic medical conditions, longer hospitalization and cost of treatment as shown by Nadiger et al. (30).

In our study, there was a bimodal pattern of seasonality with peaks during the first dry season and the short rainy season for rhinovirus, RSV and adenovirus, unlike that seen in previous studies from similar regions (6, 18, 20). This first dry season also coincides with the school reopening after a long holiday, thus increased risk of infection as schools serve as high transmission settings (31). Seasonal variations particularly with RSV have been described in previous studies conducted in Kenya. Awori et al. (2) study showed two seasonal peaks for RSV and metapneumovirus between January to April and October to December, (first dry season and the short rainy season), however rhinovirus appeared to be perennial (2). The bacterial infections peaked between January and March during the first dry season.

Berkley et al. reported that more than 50% of pneumonia cases with RSV were also detected in the long dry season (December to March). They also noted that radiologically confirmed pneumonia varied substantially by season, with a peak in November to January (short rainy season) and a trough in April to June (long rainy season) (4). This would also be an ideal time to administer RSV vaccines or the monoclonal antibody, Nirsevimab if available, to mothers at risk of preterm deliveries between 32 and 36 weeks of gestation and to preterm babies and neonates respectively (32). Both influenza A and Influenza B were more prevalent in the under 5-year age group and tended to peak just before the start of the short rainy season. Influenza vaccination of children under five years of age as well as those with chronic conditions can be intensified during the trough period between April to September.

WHO defines any hospitalised case with respiratory symptoms and fever as a SARI. Whilst this was meant to be used for influenza surveillance it has low sensitivity for detection of it and other viruses (33). We integrated our national paediatric guidelines to further classify the clinical severity of our patients into mild, moderate and severe, based on the level of oxygen need and the type of respiratory support demands which determined the level of care within the hospital^1^. In addition, we identified cases based on known diagnostic WHO ICD-10 terms for respiratory tract infections^2.^

Our five-year study period was wide and provided additional new regional data on atypical bacterial pathogens and viruses, before and after the COVID-19 era that includes clinical severity, associated factors, outcome and seasonality of the different pathogens. This aids in making a presumptive aetiological diagnosis about the pathogens causing SARIs in cases where there is inaccessibility to the tests and prognosis based on severity. Seasonality data also helps plan healthcare services, to meet the expected rise in burden during peak periods.

We acknowledge that our study was conducted in a single private center in an urban setting and may thus have lower generalizability to the rural low-income public facilities in the region. Very few patients were subjected to the traditional diagnostic studies to identify LRTI in our study. Moreover, the emphasis on BioFire® RP2.1 *plus* may have missed diagnosis of important bacterial pathogens including the typical bacterial pathogens and may not give a complete profile of the bacterial infections in our cohort.

A well-designed prospective study including both rural and urban paediatric populations with a multiplex PCR panel comprising typical, atypical bacterial and viral pathogens together with the traditional diagnostic tests like sputum cultures performed on induced sputum would provide more clarity on the epidemiology of LRTIs in our region. However, the present high cost of the multiplex PCR panel prohibits the application of this valuable diagnostic tool in the public sector.

We attempted to link pathogens detected by BioFire® RP2.1 *plus* in nasopharyngeal samples to the etiology of pneumonia in children, but were limited by the retrospective nature of the study lacking community controls. Including appropriate community controls would have allowed us to estimate pathogen-attributable fractions, as was done in the PERCH study.The cost-effectiveness of multiplex qRT-PCR/BioFire® RP2.1 *plus* testing in our setting is challenging While this approach provided valuable aetiological and seasonal data, its relatively high cost may limit routine applicability in resource-constrained settings. Accordingly, broader implementation should be considered in the context of local resources, patient selection, and targeted testing strategies.

## 5 Conclusion

Viruses accounted for more than 90% of all the pathogens detected by multiplex PCR in children with SARI. The most common viruses detected in all age groups were rhinovirus, RSV and adenovirus. *M. pneumoniae* was the most common atypical bacteria detected. *Bordetella spp.* a vaccine-preventable atypical pathogen continues to cause severe disease in children less than five years of age in our region even with high primary vaccine coverage. Vaccines against influenza and RSV should be scheduled prior to the main peaks of September and April every year, during the trough period, between April and September.

## Data Availability

The original contributions presented in the study are included in the article/Supplementary Material, further inquiries can be directed to the corresponding author.

## Footnotes

^1^Kenya Meteorological Department. Seasonal forecast.Available from: https://meteo.go.ke/resources/downloads/seasonal-forecast. Date last updated :February 22 2022.Date last accessed: November 12 2023.

^2^ Ministry of Health Republic of Kenya. Basic paediatric protocols 5th edition 2022. Ministry of Health. Available from: http://guidelines.health.go.ke/#/category/55/478/meta.Date last updated:November 1 2022.Date last accessed:October 22 2023.

^3^International Statistical Classification of Diseases and Related Health Problems (ICD).Chapter X Diseases of the respiratory system (J00-J99).https://www.who.int/standards/classifications/classification-of-diseases. Date last updated:September 1 2020.Date last accessed: March 1 2025

^4^Kenya National Bureau of Statistics (KNBS) and ICF.Kenya Demographic and Health Survey 2022:Key Indicators Report. Nairobi, Kenya, and Rockville, Maryland, USA:KNBS and ICF;2023.Available from https://dhsprogram.com/pubs/pdf/PR143/PR143.pdf.Date last updated:January 2023.Date last accessed: November 12 2023.
