# Supplementary material for: Epidemiology of viral and atypical bacterial respiratory infections among children hospitalised at a Kenyan tertiary referral centre: a cross sectional multiplex PCR study
Source: Front Pediatr. 2026 Jul 23;14:1792031. doi: 10.3389/fped.2026.1792031 (PMC13442427; doi:10.3389/fped.2026.1792031)
Supplement: Supplementary file 1 [file Table1.docx]

Supplementary Table S1: Normal Laboratory reference ranges

| **Laboratory test** | **Normal Laboratory reference ranges** |
| --- | --- |
| **Kidney function test** | |
| Sodium | (135.00 - 145.00) mmol/L |
| Potassium | (3.40 - 5.1) mmol/L |
| Chloride | (98 – 118) mmol/L |
| Urea | (2.5- 6.40) mmol/L |
| Creatinine | (27.00 - 119.00) μmol/L |
| **Vitamin D** | |
| Deficient | (0-50) nmol/L |
| Insufficient | (50-74) nmol/L |
| Normal | (75-200) nmol/L |
| **Hemogram** | |
| White blood cells(WBCs) | (5.5 - 15.0 ) × 10^9^ cells/μL |
| Neutrophil | (1.30 - 7.50 ) × 10³ cells/μL |
| Lymphocyte | (2.50 - 9.00) × 10³ cells/μL |
| Monocyte | (0.20 - 1.00) × 10³ cells/μL |
| Eosinophil | ( 0.10 - 1.00) × 10³ cells/μL |
| Red blood cells(RBC ) | (3.90 - 5.30) × 10^12^ cells/μL |
| Hemoglobin | (11.0 - 14.0) g/dL |
| Hematocrit(HCT) | (34.0 - 40.0 ) % |
| Mean corpuscular volume (MCV) | (72.0 - 87.0 ) fL |
| Mean corpuscular hemoglobin(MCH) | (24.0 - 30.0) pg |
| Mean corpuscular hemoglobin concentration(MCHC) | (31.0 - 37.0) g/dL |
| Platelets | (150 – 450) × 10³ cells/μL |
| **Liver function tests** | |
| Alanine transaminase(ALT) | (0-34)U/L |
| Aspartate transaminase(AST) | (5-34)U/l |
| Gamma-glutamyl transferase(GGT) | (9-36)U/l |
| Alkaline Phosphatase(ALP) | (54-369)U/L |
| Albumin | (37-56)g/L |
| Total protein | (64-83)g/L |
| Globulin | (12-39)g/L |
| Total Bilirubin | (0-17.1)μmol/L |
| Direct Bilirubin | (0-8.6)μmol/L |
| **Inflammatory markers** |  |
| C-reactive protein | 0.0-5.0mg/L |
| Procalcitonin | 0.0-2.0ng/ml |

Supplementary table S2: Respiratory pathogens detected on BioFire^®^ FILMARRAY^®^ Respiratory 2.1 *plus* panel.

| **Viruses** | | **Bacteria** |
| --- | --- | --- |
| ADENOVIRUS  CORONAVIRUS 229E | INFLUENZA A  INFLUENZA A/H1 | *Bordetella pertussis* |
| CORONAVIRUS HKU1  CORONAVIRUS OC43 | INFLUENZA A/H1- 2009 | *Bordetella*  *parapertussis* |
| CORONAVIRUS NL63 | INFLUENZA A/H3 | *Chlamydophila*  *pneumoniae* |
| MIDDLE EAST RESPIRATORY SYNDROME CORONAVIRUS MERS-CoV) | INFLUENZA B  PARAINFLUENZA 1 | *Mycoplasma pneumoniae* |
| SEVERE ACUTE RESPIRATORY SYNDROME CORONAVIRUS 2 (SARS-CoV-2) | PARAINFLUENZA 2  PARAINFLUENZA 3 |  |
| HUMAN METAPNEUMOVIRUS | PARAINFLUENZA 4 |  |
| HUMAN RHINOVIRUS/ENTEROVIRUS | RESPIRATORY SYNCYTIAL VIRUS (RSV) |  |

Supplementary table S3: Distribution of the pathogens by age group and the respiratory pathogens detected by BioFire® respiratory panel 2.0 (RP 2.0) and 2.1*plus* (RP2.1*plus*) test amongst paediatric patients hospitalised between March 2018 and March 2023 at a tertiary hospital in Nairobi, Kenya.

| **PATHOGEN COUNT** | **<1MONTH** | **1 MONTH**  **-1 YEAR** | **1-5 YEARS** | **5-10 YEARS** | **>10**  **YEARS** | **TOTAL**  **N=545** |
| --- | --- | --- | --- | --- | --- | --- |
| Adenovirus | 0 | 13(2.4) | 57(10.4) | 12(2.2) | 2(0.4) | 84(15.4) |
| Corona  Virus 229E | 0 | 1(0.2) | 0 | 0 | 1(0.2) | 2(0.4) |
| Corona  Virus HKU1 | 0 | 0 | 1(0.2) | 1(0.2) | 0 | 2(0.4) |

| Corona  Virus OC43 | 0 | 3(0.6) | 9(1.6) | 2(0.4) | 0 | 14(2.5) |
| --- | --- | --- | --- | --- | --- | --- |
| Corona  Virus NL63 | 0 | 0 | 6(1.1) | 1(0.2) | 0 | 7(1.3) |
| Severe acute respiratory syndrome coronavirus 2 (SARS-CoV-2) | 0 | 12(2.2) | 6(1.1) | 2(0.4 | 0 | 20(3.7) |
| Human Metapneumovirus | 0 | 12(2.2) | 20(3.7) | 2(0.4) | 1(0.2) | 35(6.4) |
| Human Rhinovirus/ Enterovirus | 2(0.4) | 36(6.6) | 75(13.7) | 14(2.6) | 4(0.7) | 131(24) |
| Influenza A | 0 | 0 | 1(0.2) | 0 | 0 | 1(0.2) |
| Influenza A/H1 | 0 | 0 | 1(0.2) | 0 | 1(0.2) | 2(0.4) |
| Influenza A/H 1-2009 | 0 | 5(0.9) | 19(3.5) | 3(0.6) | 0 | 27(4.9) |
| Influenza A/H3 | 0 | 2(0.4) | 5(0.9) | 4(0.7) | 0 | 11(2) |
| Influenza B | 0 | 0 | 9(1.6) | 5(0.9) | 0 | 14(2.6) |
| Para  Influenza 1 | 0 | 0 | 5(0.9) | 0 | 0 | 5(0.9) |
| Para  Influenza 2 | 0 | 2(0.4) | 7(1.3) | 0 | 0 | 9(1.6) |
| Para  Influenza 3 | 0 | 9(1.6) | 18(3.3) | 0 | 1(0.2) | 28(5.1) |
| Para  Influenza 4 | 0 | 1(0.2) | 7(1.3) | 1(0.2) | 0 | 9(1.6) |
| Respiratory Syncytial Virus(RSV) | 5(0.9) | 42(7.7) | 62(11.3) | 2(0.4) | 2(0.4) | 113(20.7) |
| *Bordetella pertussis* | 1(0.2) | 3(0.6) | 4(0.7) | 1(0.2) | 1(0.2) | 10(1.8) |
| *Bordetella parapertussis* | 0 | 3(0.6) | 2(0.4) | 0 | 0 | 5(0.9) |
| *Mycoplasma pneumoniae* | 0 | 2(0.4) | 7(1.3) | 3(0.6) | 1(0.2) | 13(2.4) |
| *Chlamydia pneumoniae* | 0 | 1(0.2) | 1(0.2) | 1(0.2) | 0 | 3(0.5) |

Supplementary table S4: Distribution of pathogens detected by BioFire® Respiratory panel 2.0 (RP 2) and 2.1*plus* (RP2.1*plus*) test amongst paediatric patients hospitalised between March 2018 and March 2023 at a tertiary hospital in Nairobi, Kenya based on the nature of infection.

|  | **SINGLE**  **ORGANISM** | |  | **MULTIPLE ORGANISMS** |  | **TOTAL** |  | |
| --- | --- | --- | --- | --- | --- | --- | --- | --- |
| **MICROORGANISM** | **n=262** | | **%** | **n=283** | **%** | **N=545** | **%** | |
| Adenovirus | 36 | | 6.6 | 48 | 8.8 | 84 | 15.4 | |
| Corona  Virus 229E | 0 | | 0 | 2 | 0.4 | 2 | 0.4 | |
| Corona  Virus HKU1 | 0 | | 0 | 2 | 0.4 | 2 | 0.4 | |
| Corona  Virus OC43 | 3 | | 0.6 | 10 | 1.8 | 13 | 2.4 | |
| Corona  Virus NL63 | 2 | | 0.4 | 5 | 0.9 | 7 | 1.3 | |
| Middle East Respiratory Syndrome Coronavirus  (Mers-Cov) | 0 | | 0 | 0 | 0 | 0 | 0 | |
| Severe acute respiratory syndrome coronavirus 2 (SARS-CoV-2) | 8 | | 1.5 | 12 | 2.2 | 20 | 3.7 | |
| Human Metapneumovirus | 20 | | 3.7 | 16 | 2.9 | 36 | 6.6 | |
| Human Rhinovirus/ Enterovirus | 63 | | 11.6 | 68 | 12.5 | 131 | 24 | |
| Influenza A | 0 | | 0 | 1 | 0.2 | 1 | 0.2 | |
| Influenza A/H1 | 2 | | 0.4 | 0 | 0 | 2 | 0.4 | |
| Influenza A/H 1-2009 | 18 | | 3.3 | 9 | 1.7 | 27 | 5.0 | |
| Influenza A/H3 | 7 | | 1.3 | 4 | 0.7 | 11 | 2 | |
| Influenza B | 7 | | 1.3 | 7 | 1.3 | 14 | 2.6 | |
| Para  Influenza 1 | 2 | | 0.4 | 3 | 0.5 | 5 | 0.9 | |
| Para  Influenza 2 | 1 | | 0.2 | 8 | 1.5 | 9 | 1.7 | |
| Para  Influenza 3 | 6 | | 1.1 | 22 | 4 | 28 | 5.1 | |
| Para  Influenza 4 | 4 | | 0.7 | 5 | 0.9 | 9 | 1.6 | |
| Respiratory Syncytial Virus (RSV) | 65 | 1.9 | | 48 | 8.8 | 113 | 20.7 |  |
| *Bordetella pertussis* | 3 | 0.5 | | 7 | 1.3 | 10 | 1.8 |  |
| *Bordetella parapertussis* | 1 | 0.2 | | 4 | 0.7 | 5 | 0.9 |  |
| *Chlamydophila pneumoniae* | 3 | 0.6 | | 0 | 0 | 3 | 0.6 |  |
| *Mycoplasma pneumoniae* | 11 | 2 | | 2 | 0.4 | 13 | 2.4 |  |

Supplementary table S5: Haematological parameters and severity of the respiratory infections amongst paediatric patients hospitalised between March 2018 and March 2023 at a tertiary hospital in Nairobi, Kenya.

|  | **MILD** | | **MODERATE** | | | **SEVERE** | | **TOTAL** | | ***P-***  **VALUE** |
| --- | --- | --- | --- | --- | --- | --- | --- | --- | --- | --- |
| **HAEMOGLOBIN** |  | | | | | | | | | |
| Normal | 221(58.0%) | | 77(20.2%) | | | 9(2.4%) | | 307(80.6%) | | 0.818 |
| Elevated | 54(14.2%) | | 15(3.9%) | | | 3(0.8%) | | 72(18.9%) | |  |
| Low | 2(0.5%) | | 0(0) | | | 0(0) | | 2(0.5%) | |  |
| Total | 277(72.7%) | | 92(24.1%) | | | 12(3.1%) | | 381(100%) | |  |
| **WBCS** |  | | | | | | | | | |
| Normal | 188(49.3%) | | 60(15.8%) | | | 7(1.8%) | | 255(66.9%) | | 0.315 |
| Elevated | 59(15.5%) | | 27(6.3%) | | | 4(1.0%) | | 90(23.6%) | |  |
| Low | 30(7.9%) | | 5(1.3%) | | | 1(0.3%) | | 36(9.4%) | |  |
| Total | 277(72.7%) | | 92(24.1%) | | | 12(3.1%) | | 381(100%) | |  |
| **NEUTROPHILS** |  | | | | | | | | | |
| Normal | 147(38.6%) | | 54(14.2%) | | | 8(2.1%) | | 209(54.9%) | | 0.573 |
| Elevated | 98(25.7%) | | 30(7.9%) | | | 2(0.5%) | | 130(34.1%) | |  |
| Low | 32(8.4%) | | 8(2.1%) | | | 2(0.5%) | | 42(11.0%) | |  |
| Total | 277(72.7%) | | 92(24.1%) | | | 12(3.1%) | | 381(100%) | |  |
| **LYMPHOCYTES** | |  | | | | | | | | |
| Normal | | 122(32.0%) | | 32(8.4%) | 4(1.0%) | | 158(41.5%) | | 0.257 | |
| Elevated | | 24(6.3%) | | 12(3.1) | 0(0) | | 36(9.4%) | |  | |
| Low | | 131(34.4%) | | 48(12.6%) | 8(2.1%) | | 187(49.1%) | |  | |
| Total | | 277(72.7%) | | 92(24.1%) | 12(3.1%) | | 381(100%) | |  | |
| **MONOCYTES** | |  | | | | | | | | |
| Normal | | 172(45.1%) | | 59(15.5%) | 4(1.0%) | | 235(61.7%) | | 0.055 | |
| Elevated | | 90(23.6%) | | 27(7.1%) | 5(1.3%) | | 122(32.0%) | |  | |
| Low | | 15(3.9%) | | 6(1.6%) | 3(0.8%) | | 24(6.3%) | |  | |
| Total | | 277(72.7%) | | 92(24.1%) | 12(3.1%) | | 381(100%) | |  | |
| **EOSINOPHILS** | |  | | | | | | | | |
| Normal | | 88(23.1%) | | 32(8.4%) | 3(0.8%) | | 123(32.3%) | | 0.513 | |
| Elevated | | 6(1.6%) | | 4(1.0%) | 1(0.3%) | | 11(2.9%) | |  | |
| Low | | 183(48.0%) | | 56(14.7%) | 8(2.1%) | | 247(64.8%) | |  | |
| Total | | 277(72.7%) | | 92(24.1%) | 12(3.1%) | | 381(100%) | |  | |
| **PLATELETS** | |  | | | | | | | | |
| Normal | | 204(53.5%) | | 66(17.3%) | 9(2.4%) | | 279(73.2%) | | 0.836 | |
| Elevated | | 65(17.1%) | | 23(6.0%) | 2(0.5%) | | 90(23.6%) | |  | |
| Low | | 8(2.1%) | | 3(0.8%) | 1(0.3%) | | 12(3.1%) | |  | |
| Total | | 277(72.7%) | | 92(24.1%) | 12(3.1%) | | 381(100%) | |  | |

Abbreviations: WBCs, white blood cells. *p*-values by Chi^2^ for binary/categorical variables

Supplementary table S6: Liver and kidney function test results amongst paediatric patients hospitalised with respiratory infections between March 2018 and March 2023 at a tertiary hospital in Nairobi, Kenya

| **BLOOD TEST** | **ABNORMAL** | **NORMAL** | **TOTAL** |
| --- | --- | --- | --- |
| Liver function test | 24(80%) | 6(20%) | 30(100.0%) |
| Kidney function test | 101(52.9%) | 90(47.1%) | 191(100.0%) |

Supplementary table S7: Infection markers and severity of the respiratory infection amongst paediatric patients hospitalised between March 2018 and March 2023 at a tertiary hospital in Nairobi, Kenya

|  | **MILD** | **MODERATE** | **SEVERE** | **TOTAL** | ***P-*VALUE** |
| --- | --- | --- | --- | --- | --- |
| **CRP LEVEL** |  | | | | |
| Normal | 101(28.1%) | 41(11.4%) | 5(1.4%) | 147(40.9%) | 0.602 |
| Elevated | 156(42.5%) | 50(13.9%) | 6(1.7%) | 212(59.1%) |  |
| Total | 257(71.6%) | 91(25.3%) | 11(3.1) | 359(100%) |  |
| **PROCALCITONIN** |  | | | | |
| Normal | 78(54.5%) | 31(21.7%) | 4(2.8%) | 113(79.0%) | 0.994 |
| Elevated | 21(14.7%) | 8(5.6%) | 1(0.7%) | 30(21.0%) |  |
| Total | 99(69.2%) | 39(27.3%) | 5(3.5%) | 143(100%) |  |

Abbreviations: CRP, C-reactive protein. *p*-values by Chi^2^ for binary/categorical variables.
